# Casein Kinase 1 Family Member CK1δ/Hrr25 Is Required for Autophagosome Completion

**DOI:** 10.3389/fcell.2020.00460

**Published:** 2020-07-07

**Authors:** Yuting Li, Xuechai Chen, Qianqian Xiong, Yong Chen, Hongyu Zhao, Muhammad Tahir, Jingdong Song, Bing Zhou, Juan Wang

**Affiliations:** ^1^College of Life Science and Bioengineering, Beijing University of Technology, Beijing, China; ^2^National Laboratory of Biomacromolecules, CAS Center for Excellence in Biomacromolecules, Institute of Biophysics, Chinese Academy of Sciences, Beijing, China; ^3^National Institute for Viral Disease Control and Prevention, Chinese Center for Disease Control and Prevention, Beijing, China; ^4^State Key Laboratory of Membrane Biology, School of Life Sciences, Tsinghua University, Beijing, China

**Keywords:** autophagy, CK1δ, Hrr25, isolation membrane, autophagosome closure

## Abstract

Autophagy starts with the initiation and nucleation of isolation membranes, which further expand and seal to form autophagosomes. The regulation of isolation membrane closure remains poorly understood. CK1δ is a member of the casein kinase I family of serine/threonine specific kinases. Although CK1δ is reported to be involved in various cellular processes, its role in autophagy is unknown. Here, we show that CK1δ regulates the progression of autophagy from the formation of isolation membranes to autophagosome closure, and is essential for macroautophagy. CK1δ depletion results in impaired autophagy flux and the accumulation of unsealed isolation membranes. The association of LC3 with ATG9A, ATG14L, and ATG16L1 was found to be increased in CK1δ-depleted cells. The role of CK1δ in autophagosome completion appears to be conserved between yeasts and humans. Our data reveal a key role for CK1δ/Hrr25 in autophagosome completion.

## Introduction

Autophagy is an evolutionarily conserved pathway in which cytoplasmic components are sequestered within double-membrane vesicles called autophagosomes, and then transported into lysosomes or vacuoles for degradation (Nakatogawa et al., 2009; Lamb et al., 2013; Feng et al., 2014). Autophagy is essential for cellular homeostasis and the cellular response to stress conditions such as nutrient starvation. Defects in autophagy pathways have been associated with numerous human pathologies including infectious diseases, neurodegenerative disorders, and cancer (Yang and Klionsky, 2010; Dikic and Elazar, 2018). Starvation-induced macroautophagy is non-selective; by contrast, selective autophagy involves the recognition of cellular cargoes by specific receptors and their subsequent engulfment by autophagosomes (Nakatogawa et al., 2009; Lamb et al., 2013; Feng et al., 2014).

Autophagy is initiated with the *de novo* formation of a cup-shaped membrane, known as the isolation membrane or phagophore, which expands and seals to form the autophagosome (Nakatogawa et al., 2009; Lamb et al., 2013; Feng et al., 2014). In mammalian cells, this is followed by the fusion of autophagosomes with endosomes and lysosomes to form degradative autolysosomes. Autophagy is regulated by autophagy-related (ATG) proteins, which are recruited to the site of autophagosome formation, in a hierarchical order, upon autophagy induction (Nakatogawa et al., 2009; Lamb et al., 2013; Feng et al., 2014). Autophagy-related proteins include the ULK1/Atg1 complex, which is required for the initiation of autophagy; the PI3K complex, which is essential for nucleation of the isolation membrane; Atg9, the only transmembrane core ATG protein, which is required during the early stages of autophagy; and the Atg12 and Atg8 conjugation systems, which have roles in vesicle expansion. Although most ATG proteins disassociate from the autophagic membrane structures during autophagosome closure, the lipidated form of LC3/Atg8 associates with autophagic structures at all stages; therefore, LC3/Atg8 represents a useful marker of isolation membranes and autophagosomes (Lamb et al., 2013; Klionsky et al., 2016).

CK1δ (casein kinase I δ), a member of the CK1 family of serine/threonine specific kinases, is involved in the regulation of various cellular processes including circadian rhythms, Wnt signaling, cytoskeleton maintenance, the cell cycle, and DNA damage repair (Xu et al., 2019). Hrr25, the yeast homolog of CK1δ, has been reported to activate multiple selective autophagy pathways by phosphorylating cargo receptors and promoting the interactions of these receptors with the scaffold protein Atg11 (Mochida et al., 2014; Pfaffenwimmer et al., 2014; Tanaka et al., 2014). We previously reported that Hrr25 is also required for macroautophagy (Wang et al., 2015). However the role of CK1δ in macroautophagy in mammalian cells remains unclear.

In this study, we show that CK1δ is essential for macroautophagy in mammalian cells, and that CK1δ depletion or Hrr25 mutation results in blockade of the progression of isolation membranes to autophagosomes, thus revealing a key role of CK1δ/Hrr25 in autophagosome completion.

## Materials and Methods

### Cell Culture and Transfection

HeLa cells were cultured in DMEM (Hyclone) and 10% fetal bovine serum (FBS, Gibco) supplemented with 1% penicillin-streptomycin (Gibco) at 37°C and 5% CO_2_. For starvation, cells were washed with PBS three times and incubated with Earle’s balanced salt solution (EBSS, Gibco) for 2–h at 37°C. Transfection of plasmids was carried out with Lipofectamine 3000 (Invitrogen) according to the manufacturer’s protocols. Transfection of small interfering RNAs (siRNAs) was carried out with Lipofectamine RNAi MAX (Invitrogen). To knockdown CK1δ, double-stranded siRNAs were purchased from GenePharma. The following sequences were used: human CK1δ siRNA 5′-CGACCUCACAGGCCGACAATT-3′ and control siRNA 5′-UUCUCCGAACGUGUCACGUTT-3′.

### Yeast Media

Yeast cells were grown at 25°C in yeast extract peptone dextrose media (YPD; 1% yeast extract, 2% peptone, and 2% dextrose) or synthetic minimal media (SMD; 0.67% yeast nitrogen base, 2% dextrose, and auxotrophic amino acids, as needed). To induce starvation, yeast cells were transferred to SD(–N) medium (0.17% yeast nitrogen base without amino acids and 2% dextrose) or treated with 400 ng/ml rapamycin.

### Quantitative RT-PCR

Total RNA was extracted from CK1δ-knockdown HeLa cells using the Cultured CellTotal RNA Extraction Kit (TIANGEN), and cDNA was reverse-transcribed using the FastQuant RT Kit(TIANGEN). Quantitative PCR was carried out on a Step One PlusTM RealTime PCR system using SuperReal PreMix Plus (TIANGEN). Data were normalized to the expression level of β-actin. Results are representative of at least three experiments. The following primers were used: F-CK1 Delta, 5′-CTCCGTGTTCCGTTTC-3′; R-CK1 Delta, 5′-TGCTACTCGCCATCCT-3′; F-GAPDH, 5′-GGCATCCTGGGCTACACTGA-3′; R-GAPDH, 5′-GTGGTC GTTGAGGGCAATG-3′.

### Immunoblotting

Total proteins were extracted from HeLa cells with RIPA Lysis Buffer (Solarbio) supplemented with 1 mM PMSF, and incubated for 30 min on 4°C. Cell lysates were centrifuged at 12000 *g* for 30 min at 4°C. Supernatants were separated by SDS-PAGE and transferred onto a PVDF membrane, followed by incubation with primary and secondary antibodies (Table 1); then, the PVDF membrane was visualized using an ECL kit (Millipore). Results are representative of at least three experiments. The relative levels of p62 and LC3-II were normalized to β-actin levels.

**TABLE 1:** List of antibodies used in this study.

Antibodies	Source	Identifier	Dilution
Rabbit poly clonal anti-p62	MBL	Cat#PM045	1:1000 (WB), 1:500 (IF)
Rabbit poly clonal anti-LC3B	Cell Signaling Technology	Cat#2775	1:1000 (WB)
Rabbit poly clonal anti-P-Actin	Solarbio	Cat#K101527P	1:1000 (WB)
Rabbit poly clonal anti-GFP	Abeam	Cat#ab290	1:2000 (WB)
Rabbit Poly clonal anti-Casein Kinase 1 delta	Proteintech	Cat# 14388-1-AP	1:600 (WB)
Goat Anti-Rabbit IgG-HRP	Solarbio	Cat#SE134	1:5000 (WB)
Rabbit poly clonal anti-ATG14	Cell Signaling Technology	Cat#5504	1:100 (IF)
Rabbit monoclonal anti-ATG16Ll	Cell Signaling Technology	Cat#8089	1:80 (IF)
Rabbit poly clonal anti-ATG9A	MBL	Cat#PD042	1:200 (IF)
Mouse monoclonal anti-LC3B	MBL	Cat#M152-3	1:100 (IF)
Fluorescein(FITC)-conjugated AffiniPure Goat Anti-Rabbit IgG(H+L)	Jackson Immuno Research	Cat# 111-095-003	1:100 (IF)
Flu ores cein(FITC)-conjugated AffiniPure Goat Anti -Mouse IgG(H+L)	Jackson Immuno Research	Cat# 115-095-003	1:100 (IF)
Rhodamine(TRITC)-conjugated AffiniPure Goat Anti-Mouse IgG(H+L)	Jackson Immuno Research	Cat# 115-025-003	1:100 (IF)

*WB, Western Blot; IF, Immunofluorescence.*

### Immunofluorescence

HeLa cells grown on cover slips were fixed with 4% paraformaldehyde for 20 min. Cells were permeabilized in 10 μg/mL digitonin (Sigma) for 15 min at 25°C. After blocking with 5% goat serum for 60 min at 25°C, cells were incubated with the indicated primary antibodies overnight at 4°C. After three washes with PBS, cells were stained with FITC or Rhodamine-labeled secondary antibodies for 1 h at room temperature. Nuclei were stained with 0.2% Hoechst 33258 for 30 min. Images were acquired using a FV3000 confocal microscope (Olympus). The colocalization was measured using the ImageJ software.

### Protease Protection Assay

HeLa cells were starved in EBSS for 2 h, treated with 100 nM bafilomycin A1 for 6 h, and suspended in ice-cold homogenization buffer (composed of 20 mM HEPES, 0.22 M mannitol, 0.07 M sucrose, and protease inhibitors; pH 7.4). Cells were then passed 10 times through a 27-gauge needle using a 1 ml injection syringe, and the post-nuclear supernatant (PNS) was recovered by centrifugation at 4500 *g* and at 4°C, for 10 min. The supernatant was then centrifuged again at 100,000 *g* for 30 min to acquire the pellet fraction. The pellet was resuspended in homogenization buffer and then treated with 100 μg/ml proteinase K (ProK) with or without 0.5% Triton X-100. After incubation for 20 min on ice, 10% Trichloroacetic acid (TCA) was added, and the samples were centrifuged at 15,000 *g* for 10 min. The pellet was washed with ice-cold acetone, resuspended in SDS-PAGE sample buffer, and boiled at 100°C for 10 min. Proteinase K digestion products were detected by immunoblotting.

### Super-Resolution Structured Illumination Microscopy

To analyze phagophore formation, yeast cells were fixed with 3.7% formaldehyde at 25°C for 30 min, and then visualized by super-resolution structured illumination microscopy (SIM), at 25°C, on an Applied Precision DeltaVision OMX Super Resolution System using an Olympus UPlanSApo 100 × 1.4 NA oil objective. The data were acquired and processed using Delta Vision OMX Master Control software and SoftWoRx reconstruction and analysis software.

### Statistical Analysis

The significance of differences was evaluated by an unpaired two-tailed *t* test. Data were analyzed for statistical significance after at least three repeated experiments. Threshold for statistical significance for each test was set at 95% confidence (*p* < 0.05). Error bars represent SEM. ^∗^*p* < 0.05, ^∗∗^*p* < 0.01. ^∗∗∗^*p* < 0.001.

## Results

### Depletion of CK1δ Impairs Autophagic Flux

**FIGURE 1:**
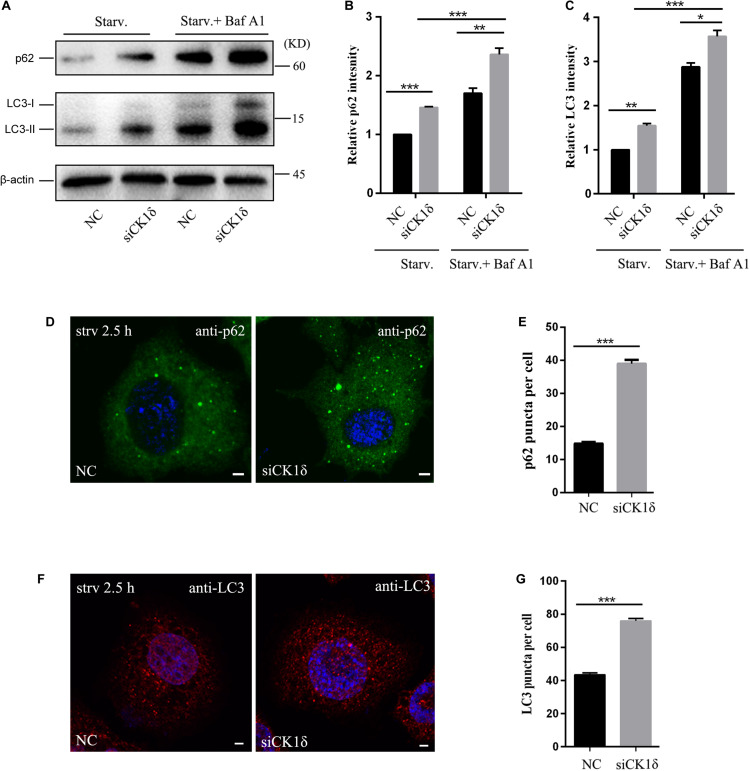
CK1δ depletion impairs autophagic flux. **(A–C)** Immunoblotting of LC3 and p62 in negative control (NC) and CK1δ-depleted HeLa cells. Cells were starved were starved in EBSS for 2.5 h with or without bafilomycin A1 (100 nM, 6 h). The relative levels of p62 and LC3-II were normalized to β-actin levels as shown in panels **(B)** and **(C)**, respectively. NC under starvation condition was normalized to be 1. Error bars represent SEM; *n* = 3; *p < 0.05, **p < 0.01, ***p < 0.001, Student’s *t* test. **(D,E)** Immunostaining of p62 using endogenous antibody. CK1δ-depleted HeLa cells and control cells were starved in EBSS for 2.5 h. Scale bars, 5 μm. Quantification of p62 puncta number is shown in panel **(E)**. Cells from three separate experiments (200 in total) were examined to calculate the numbers of p62 puncta. Error bars represent SEM; ***p < 0.001, Student’s *t* test. **(F,G)** Immunostaining of LC3 using endogenous antibody. CK1δ-depleted HeLa cells and control cells were starved in EBSS for 2.5 h. Scale bars, 5 μm. Quantification of p62 puncta number is shown in panel **(G)**. Cells from three separate experiments (200 in total) were examined to calculate the numbers of LC3 puncta. Error bars represent SEM; ***p < 0.001, Student’s *t* test.

To determine whether CK1δ is essential for autophagy, we used siRNAs to deplete HeLa cells of CK1δ, and examined the autophagic flux by monitoring the level of p62, an autophagy substrate (Klionsky et al., 2016). As shown in Supplementary Figure S1, CK1δ was efficiently depleted. CK1δ depletion resulted in increased levels of p62 (Figures 1A,B). Immunostaining of p62 also showed that, the number of p62 puncta was higher in CK1δ-depleted cells than that in control cells under starvation conditions (Figures 1D,E), suggesting the blockade of autophagic flux.

Following the induction of autophagy, the non-lipidated form of LC3 (LC3-I) is conjugated to PE to form the lipidated LC3-II form, which associates with autophagic membrane structures and forms punctate structures. Analysis of LC3-II levels serves as a useful assay to determine which step of autophagy is affected (Klionsky et al., 2016). We examined the levels of LC3 II in CK1δ-depleted cells under starvation conditions with or without bafilomycin A1, an autophagosome-lysosome fusion inhibitor. As shown in Figures 1A,C, CK1δ depletion resulted in increased levels of LC3-II, indicating that the defect in autophagic flux occurs after LC3 lipidation. The addition of bafilomycin A1 significantly increased the LC3-II levels in CK1δ-depleted cells, suggesting that autophagosome-lysosome fusion is not blocked by CK1δ depletion. LC3 immunostaining revealed that the number of LC3 puncta was increased in CK1δ-depleted cells (Figures 1F,G). The non-lipidated form of LC3-I is diffuse, and only the lipidated LC3-II form associates with autophagic membrane structures; therefore, these data indicate that LC3-II-positive structures accumulate in CK1δ-depleted cells. These data are consistent with results in temperature-sensitive yeast *hrr25-5* mutant cells; in a previous study, we observed numerous punctate GFP-Atg8 structures in the *hrr25-5* mutant, despite the decreased autophagic activity in these cells (Wang et al., 2015).

Collectively, these results indicate that CK1δ depletion impairs autophagic flux downstream of LC3 lipidation and leads to the accumulation of LC3-II-positive structures.

### Unsealed Autophagosomes Accumulate in CK1δ-Depleted Cells and the *hrr25-5* Mutant

**FIGURE 2:**
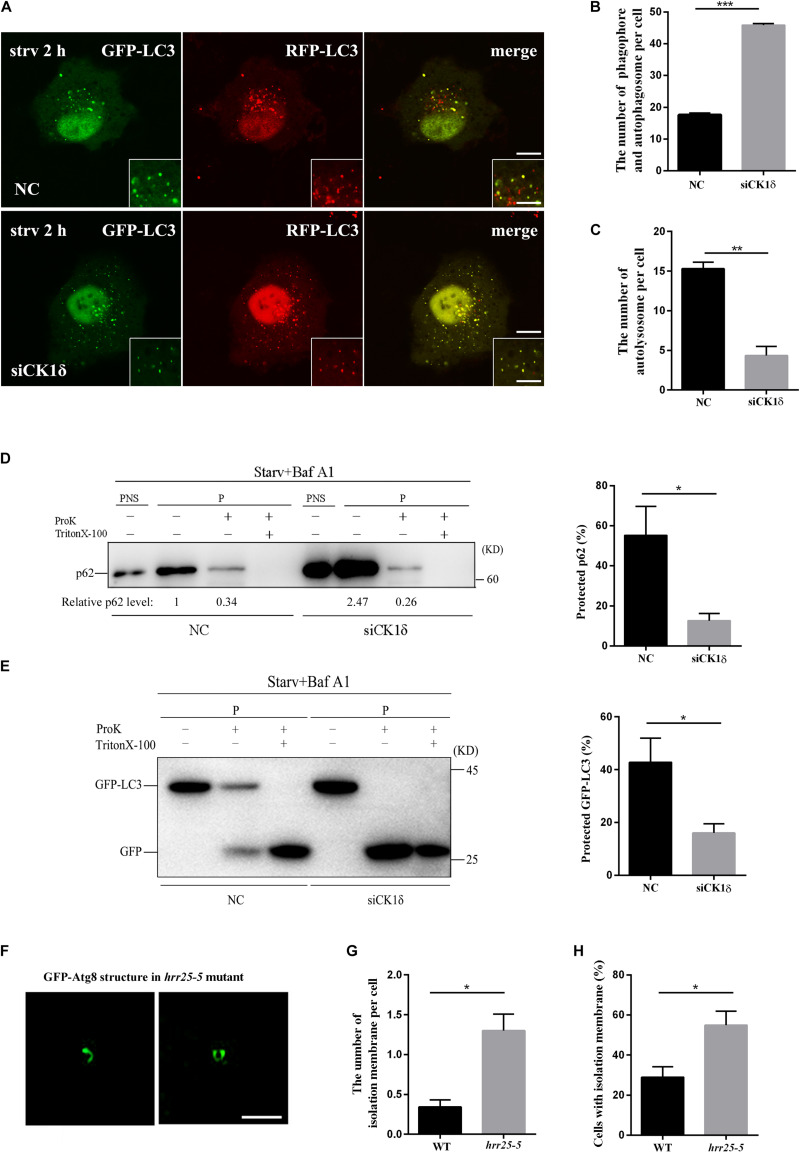
Unsealed isolation membranes accumulate in CK1δ-depleted cells and the *hrr25-5* mutant. **(A–C)** CK1δ-depleted HeLa cells and control cells transfected with RFP-GFP-LC3 reporter plasmid were starved for 2 h. Scale bars in panel **(A)**, 5 μm; Scale bars in insets, 2 μm. Quantification of the number of yellow puncta (autophagosome and isolation membranes) is shown in panel **(B)**. Quantification of the number of red puncta (autolysosome) is shown in panel **(C)**. Cells from three separate experiments (200 in total) were examined to calculate the numbers of LC3 puncta. Error bars represent SEM; **p < 0.01. ***p < 0.001, Student’s *t* test. **(D)** CK1δ-depleted HeLa cells and control cells were starved starved in EBSS for 2 h and treated with 100 nM bafilomycin A1 for 6 h. The post-nuclear supernatant (PNS) and high-speed pellet (P) fractions were analyzed by immunoblotting using anti-p62 antibody. The subfractions were incubated in the presence or absence of proteinase K (Pro K) and Triton X-100. The percentage of protected p62 were calculated from four separate experiments and shown in the right. Error bars represent SEM; *p < 0.05, Student’s *t* test. **(E)** GFP-LC3 expressing CK1δ-depleted HeLa cells and control cells were starved in EBSS for 2 h and treated with 100 nM bafilomycin A1 for 6 h. The high-speed pellet (P) fractions were analyzed by immunoblotting using anti-GFP antibody. The subfractions were incubated in the presence or absence of proteinase K (Pro K) and Triton X-100. The percentage of protected GFP-LC3 were calculated from four separate experiments and shown in the right. Error bars represent SEM; *p < 0.05, Student’s *t* test. **(F–H)** Wild-type yeast cells and *hrr25-5* mutant cells expressing GFP-Atg8 were grown to log phase and treated with 400 ng/ml rapamycin for 1 h at 37°C. Deconvolved images of isolation membranes in the *hrr25-5* mutant are shown in panel **(F)**. Scale bars, 1 μm. Numbers of isolation membranes were calculated in 100 cells from three separate experiments and shown in panel **(G)**. Percentage of cells with isolation membrane was calculated in 100 cells from three separate experiments and shown in panel **(H)**. Error bars represent SEM; *p < 0.05, Student’s *t* test.

To determine which step of the autophagy pathway is affected by CK1δ depletion, we examined the stage of the autophagic structures observed in CK1δ-depleted cells and *hrr25-5* mutant cells. The tandem red fluorescent protein (RFP)-green fluorescent protein (GFP)-LC3 reporter was transfected into CK1δ-depleted cells and control cells as previously described. The GFP fluorescence signal is quenched in acidified compartments; accordingly, prior to fusion with the lysosome, the isolation membranes and immature autophagosomes decorated with RFP-GFP-LC3 are visible as yellow puncta, whereas red puncta represent acidified autolysosomes (Kimura et al., 2007). We found that after 2 h of starvation, larger numbers of yellow puncta had accumulated and very few red puncta had formed in CK1δ-depleted cells (Figures 2A–C). These results indicate that CK1δ depletion causes a defect in the progression of isolation membranes to autolysosomes, and that accumulated LC3 puncta in CK1δ-depleted cells represent isolation membranes or autophagosomes, not autolysosomes.

To verify that the LC3-positive structures in CK1δ-depleted cells represented isolation membranes or autophagosomes, we performed a protease protection assay, which is based on the accessibility of GFP-LC3 or p62, sequestered in autophagosomes or not, to protease (Klionsky et al., 2016): when GFP-LC3 and p62 are sequestered by sealed autophagosomes, they are inaccessible to the protease; by contrast, GFP-LC3 and p62 in isolation membranes are accessible to the enzyme. We found that the sensitivity of GFP-LC3 and p62 to ProK was enhanced in CK1δ-depleted cells (Figures 2D,E). These results confirm that the LC3-positive structures in CK1δ-depleted cells are isolation membranes, revealing a role for CK1δ in autophagosome completion.

Consistent with the results in CK1δ-depleted cells, SIM revealed that elongated isolation membranes were accumulated in the temperature-sensitive *hrr25-5* mutant at 37°C, a non-permissive temperature. As shown in Figures 2F,G, the number of isolation membranes was significantly increased in the *hrr25-5* mutant compared with that in the wild type. The percentage of cells with isolation membrane was also significantly higher in the *hrr25-5* mutant (Figure 2H). Together, these findings show that CK1δ depletion or the *hrr25* mutation results in a defect in the progression of the autophagy pathway from the formation of isolation membranes to sealed autophagosomes.

### Depletion of CK1δ Results in Increased Association of LC3 With Multiple ATG Proteins

**FIGURE 3:**
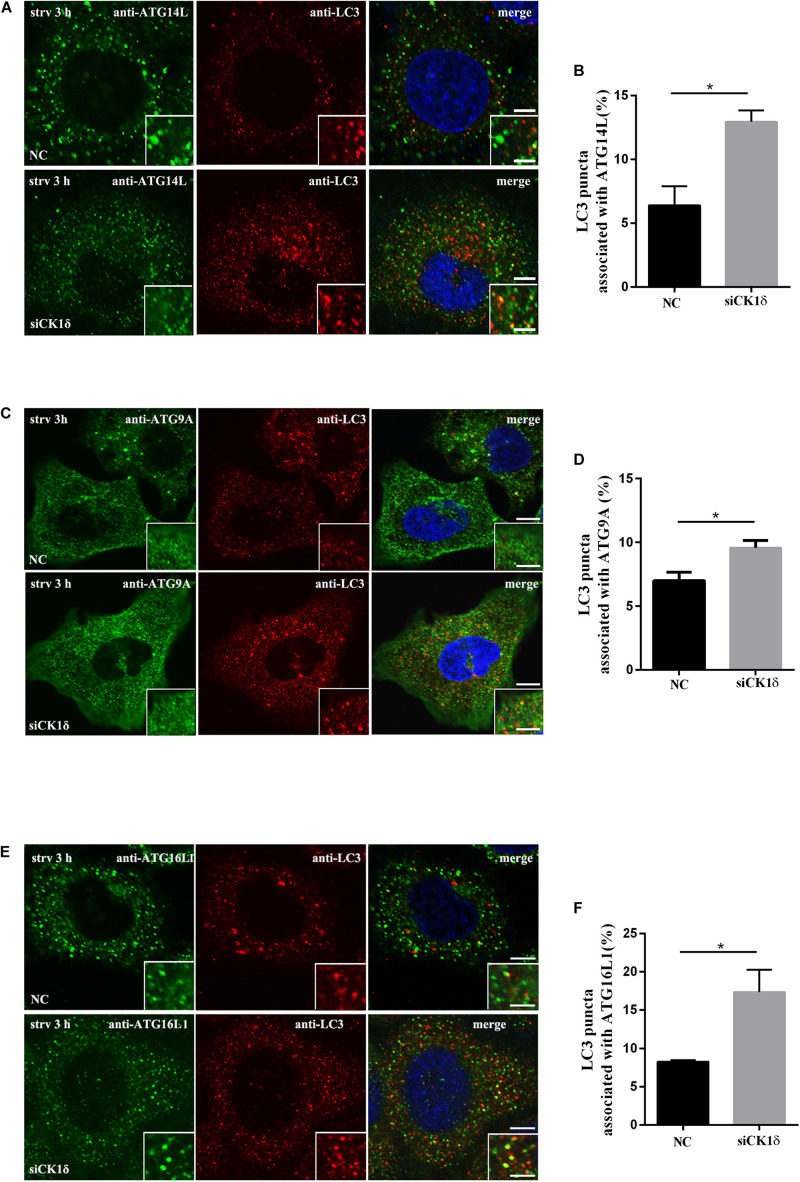
Depletion of CK1δ results in increased association of LC3 and multiple ATG proteins. **(A,B)** Immunostaining of LC3 and ATG14L using endogenous antibodies. HeLa cells depleted of CK1δ and control HeLa cells were starved for 3 h. Scale bars, 5 μm. Scale bars in insets, 2 μm. Quantification of the percentage of LC3 puncta that associate with ATG14L puncta is shown in panel **(B)**. Cells from three separate experiments (150 in total) were examined. Error bars represent SEM; *p < 0.05, Student’s *t* test. **(C,D)** Immunostaining of LC3 and ATG9A using endogenous antibodies. HeLa cells depleted of CK1δ and control HeLa cells were starved for 3 h. Scale bars, 5 μm. Scale bars in insets, 2 μm. Quantification of the percentage of LC3 puncta that associate with ATG9A puncta is shown in panel **(D)**. Cells from three separate experiments (150 in total) were examined. Error bars represent SEM; *p < 0.05. Student’s *t* test. **(E,F)** Immunostaining of LC3 and ATG16L1 using endogenous antibodies. HeLa cells depleted of CK1δ and control HeLa cells were starved for 3 h. Scale bars, 5 μm. Scale bars in insets, 2 μm. Quantification of the percentage of LC3 puncta that associate with ATG16L1 puncta is shown in panel **(D)**. Cells from three separate experiments (150 in total) were examined. Error bars represent SEM; *p < 0.05. Student’s *t* test.

Upon autophagy induction, ATG proteins are recruited to the autophagosome formation site in a hierarchical order. Most ATG proteins disassociate from the autophagic membrane structures as autophagosomes close, whereas the lipidated form of LC3/Atg8 associates with autophagic structures at all stages (Itakura and Mizushima, 2010; Lamb et al., 2013). Failure of isolation membranes to seal and form closed autophagosomes results in ATG proteins, which act during the early stages of autophagy, becoming trapped in the isolation membranes along with LC3. Accordingly, we examined the colocalization or association of LC3 puncta with multiple ATG proteins, including ATG9A, ATG14L, and ATG16L1, by immunofluorescence. As shown in Figure 3, the association of LC3 with all three ATG proteins increased in CK1δ-depleted cells although the numbers of ATG9A, ATG14L, and ATG16L1 puncta in CK1δ-depleted cells were comparable to those in control cells (Supplementary Figure S2). These data are consistent with the hypothesis that CK1δ regulates autophagosome completion.

## Discussion

We showed that CK1δ regulates autophagosome completion, and is required for autophagy in mammalian cells. CK1δ depletion was found to result in the accumulation of isolation membranes and increased association of LC3 with ATG9A, ATG14L, and ATG16L1. To the best of our knowledge, this is the first report of the essential role of CK1δ in macroautophagy. The role of CK1δ/Hrr25 in autophagosome completion appears to be conserved between yeasts and humans.

Although the regulation of autophagosome completion is not fully understood, several proteins have been reported to regulate autophagosome completion. In mammalian cells, depletion of CHMP2A, a component of ESCRT (endosomal sorting complex required for transport), a dominant-negative VPS4A^E228Q^ mutant, overexpression of Atg4B^C74A^ mutant, and knockdown of ATG2A and ATG2B cause defects in autophagosome closure (Fujita et al., 2008; Velikkakath et al., 2012; Takahashi et al., 2018). In yeast, the Vps21 module, ESCRT components are considered to play roles in autophagosome closure and PI3P phosphatase Ymr1 is essential for the clearance of PI3P and release of Atg proteins from the closed autophagosome (Cebollero et al., 2012; Zhou et al., 2017, 2019a,b). At present, the relationship between CK1δ and other regulators remains unknown, as do the substrates that CK1δ phosphorylates during autophagosome completion. Elucidation of these aspects should further advance the understanding of the mechanisms involved in autophagosome completion. Furthermore, the dysregulation of CK1δ and mutations in this kinase are linked to various diseases, including cancer, neurodegenerative disorders, and metabolic diseases (Xu et al., 2019); therefore, future studies should aim to determine whether CK1δ contributes to the development of these diseases through its role in autophagy.

## Data Availability Statement

All datasets generated for this study are included in the article/Supplementary Material.

## Author Contributions

JW designed the research. YL, QX, XC, YC, HZ, and JS performed the experiments. JW, YL, BZ, and QX analyzed the data. JW and MT wrote the manuscript. All authors contributed to the article and approved the submitted version.

## Conflict of Interest

The authors declare that the research was conducted in the absence of any commercial or financial relationships that could be construed as a potential conflict of interest.
