# Supplementary material for: Casein Kinase 1 Family Member CK1δ/Hrr25 Is Required for Autophagosome Completion
Source: Front Cell Dev Biol. 2020 Jul 7;8:460. doi: 10.3389/fcell.2020.00460 (PMC7381346; doi:10.3389/fcell.2020.00460)
Supplement: Supplementary file 1 [file Image_1.pdf]

Supplementary Material

Supplementary Figures

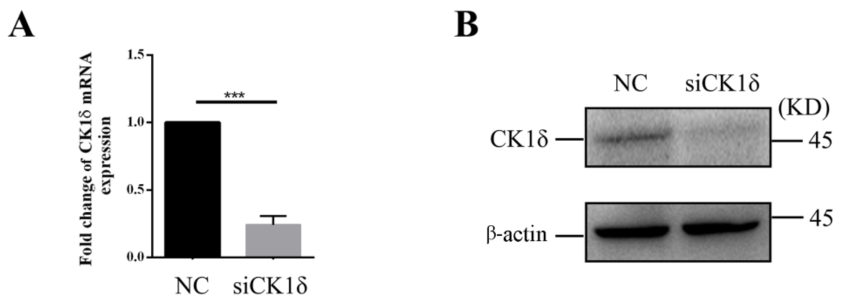

Figure S1. CK1δ was efficiently depleted by small interfering RNA.

(A) Quantitative real-time PCR analysis of negative control (NC) and CK1δ-depleted HeLa cells. Error bars represent SEM; n=3. ***, $p < 0.001$, Student’s *t* test.
(B) Immunoblotting of CK1δ in negative control (NC) and CK1δ-depleted HeLa cells.

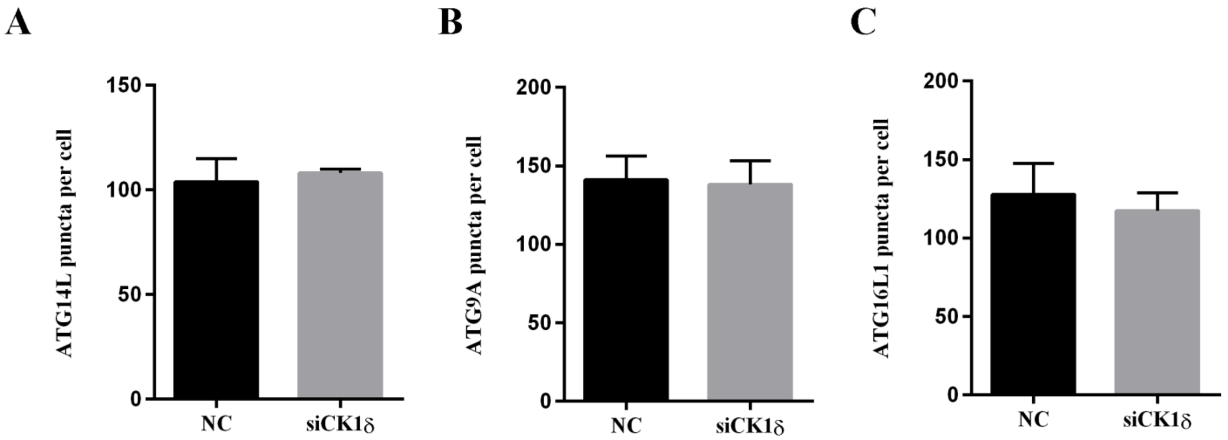

Figure S2. CK1δ depletion did not affect the number of ATG14L puncta, ATG9A puncta or ATG16L puncta.

(A) HeLa cells depleted of CK1δ and control HeLa cells were starved in EBSS for 3 h. The ATG14L puncta number was examined by immunostaining with endogenous antibody. Cells from three separate experiments (150 in total) were examined. Error bars represent SEM.

(B) HeLa cells depleted of CK1δ and control HeLa cells were starved in EBSS for 3 h. The ATG9A puncta number was examined by immunostaining with endogenous antibody. Cells from three separate experiments (150 in total) were examined. Error bars represent SEM.

(C) HeLa cells depleted of CK1δ and control HeLa cells were starved in EBSS for 3 h. The ATG16L1 puncta number was examined by immunostaining with endogenous antibody. Cells from three separate experiments (150 in total) were examined. Error bars represent SEM.
